# The Pathogenic Mechanism of *Enterocytozoon hepatopenaei* in *Litopenaeus vannamei*

**DOI:** 10.3390/microorganisms12061208

**Published:** 2024-06-15

**Authors:** Rongrong Ma, Bo Zhu, Jinbo Xiong, Jiong Chen

**Affiliations:** 1 State Key Laboratory for Managing Biotic and Chemical Threats to the Quality and Safety of Agro-Products, Ningbo University, Ningbo 315211, China; marongrong@nbu.edu.cn (R.M.); 18858444540@163.com (B.Z.); xiongjinbo@nbu.edu.cn (J.X.); 2 School of Marine Sciences, Ningbo University, Ningbo 315211, China

**Keywords:** *Enterocytozoon hepatopenaei*, *Litopenaeus vannamei*, pathogenicity, energy metabolism

## Abstract

*Enterocytozoon hepatopenaei* (EHP) is a parasite in shrimp farming. EHP mainly parasitizes the hepatopancreas of shrimp, causing slow growth, which severely restricts the economic income of shrimp farmers. To explore the pathogenic mechanism of EHP, the host subcellular construction, molecular biological characteristics, and mitochondrial condition of *Litopenaeus vannamei* were identified using transmission electron microscopy (TEM), real-time qPCR, an enzyme assay, and flow cytometry. The results showed that EHP spores, approximately 1 μm in size, were located on the cytoplasm of the hepatopancreas. The number of mitochondria increased significantly, and mitochondria morphology showed a condensed state in the high-concentration EHP-infected shrimp by TEM observation. In addition, there were some changes in mitochondrial potential, but apoptosis was not significantly different in the infected shrimp. The qPCR results showed that the gene expression levels of hexokinase and pyruvate kinase related to energy metabolism were both upregulated in the diseased *L. vannamei*. Enzymatic activity showed hexokinase and lactate dehydrogenase were significantly increased in the shrimp infected with EHP, indicating EHP infection can increase the glycolysis process and decrease the oxidative phosphorylation process of *L. vannamei*. Previous transcriptomic data analysis results also support this conclusion.

## 1. Introduction

Aquaculture is a major source of high-quality protein for meeting human demand. Among aquaculture species, *Litopenaeus vannamei* is one of the most widely farmed crustacean species worldwide [1]. With increasing market consumption, intensive high-density cultivation modes for *L. vannamei* were developed. However, high-density farming is also accompanied by frequent diseases, which lead to massive mortality of farmed shrimp and hinder the development of the shrimp industry [2]. *Enterocytozoon hepatopenaei* (EHP) belongs to microsporidia and parasitizes the hepatopancreatic epithelial cells of *L. vannamei* [3,4]. This parasite mainly causes growth retardation and exhibits no specific clinical symptoms, which usually results in depleted feed of diseased *L. vannamei*, further leading to great economic losses [5]. Although there are numerous detection methods available, a thorough understanding of the disease’s pathogenic mechanisms and effective prevention strategies are still lacking. This often results in a challenging situation where no direct measures can be taken when the emergence of the disease occurs [6,7].

Oxidative phosphorylation and glycolysis are the main pathways for energy production in eukaryotic organisms. However, a large set of core eukaryotic genes were jettisoned early in the evolutionary history of microsporidia. That made them lose the mitochondrial genome and the ability to generate ATP via oxidative phosphorylation [8]. With the absence of oxidative phosphorylation in microsporidia, glucose is only partially metabolized via glycolysis to release 7% of its full ATP potential. And energy supply is complemented by the import of ATP molecules from the host with the help of horizontally acquired ATP/ADP translocases [8,9]. Microsporidia are mainly dependent on the host cell for metabolism and energy requirements [10]. In *L. vannamei*, energy theft by EHP causes significant growth retardation.

In order to study the energy imbalance of *L. vannamei* infected by EHP, *L. vannamei* with different EHP infection levels were collected, and energy metabolism and mitochondria characteristics of *L. vannamei* infected by EHP were explored. These findings can provide some basic information for pathogenic mechanisms and further target control research.

## 2. Materials and Methods

### 2.1. Sample Collection

*L. vannamei* showed retarded growth and large individual differences in the same pond in one shrimp farming factory located in Ningbo City. To explore the reasons, shrimp samples were collected from different ponds, and the length of the shrimp was recorded. Meanwhile, a PCR-amplified molecular assay was used to detect potential pathogen infection. Shrimp infected with EHP were selected and brought back to the pilot base of Ningbo University, where they were temporarily kept in 150 L aquaculture tanks with recirculating seawater at 25 °C for the subsequent experiment.

### 2.2. Histopathological Analysis

**Figure 1:**
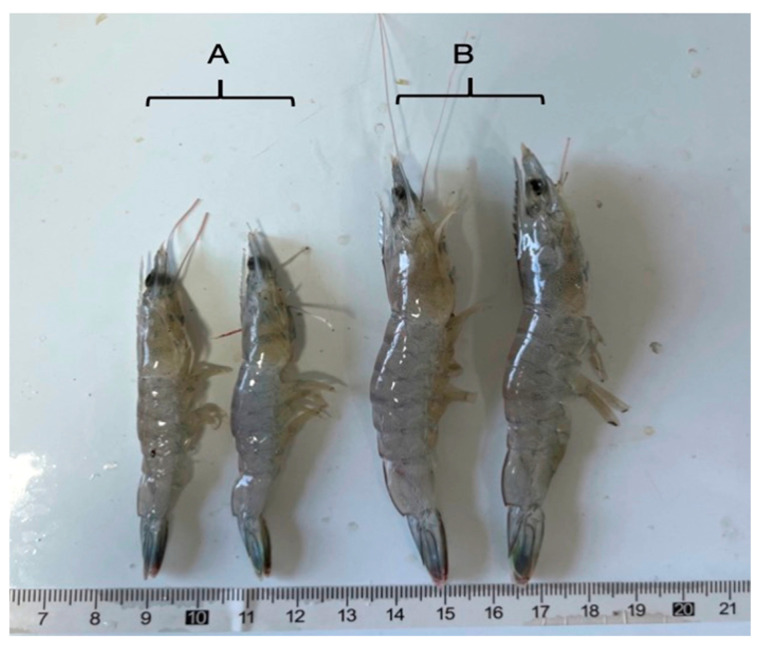
Shrimp with different concentrations of EHP infection. Note: A is the shrimp sample with a high concentration of EHP infection; B is the shrimp sample with a low concentration of EHP infection.

After fluorescence quantification of EHP pathogens, *L. vannamei* naturally infected with different levels of EHP (Figure 1) were selected for transmission electron microscopy observation.

The specific steps were as follows: EHP-infected shrimp hepatopancreases were quickly peeled off and fixed in 3% glutaraldehyde at 4 °C for 24 h. After washing with buffer, samples were fixed in 1% osmic acid at 4 °C for 2 h. After rinsing with PBS, samples were dehydrated with a graded ethanol series (30%, 50%, 70%, and 90%) followed by a graded acetone series (90% and 100%). After that, the samples were immersed in a mixture of epoxy resin and acetone. After drying, samples were cut with an ultra-thin microtome, and sections were stained with uranyl acetate and double-stained with lead citrate. Section observations were carried out using an H-7650 transmission electron microscope (Hitachi, Tokyo Japan).

### 2.3. EHP Artificial Infection Experiment

A batch of healthy and well-growing shrimp was purchased from another shrimp farm. The healthy shrimp group and the infected shrimp group were set. Each group with 100 shrimp was raised in separate 120 L aquariums. The infected group was fed with the hepatopancreas of the strong positive EHP-detected *L. vannamei*, while the control group was fed with healthy shrimp hepatopancreas. Both groups were fed twice a day continuously for 3 days. Samples were collected on the 7th and 14th day after infection for subsequent analyses. The relative quantification result of EHP in the sample was calculated using fluorogenic quantitative PCR with a previously reported primer [11].

### 2.4. Gene Expression Changes Related to Glucose Metabolism

In this study, the expression levels of hexokinase (HK) and pyruvate kinase (PK), the key genes in glycolysis, were detected in the diseased *L. vannamei* samples. More specifically, RNA extraction of the samples collected on the 7th and 14th days after infection was performed. After the reverse transcription, quantitative real-time PCR was used to detect the expression of HK and PK. The primer sequences are listed in Table 1.

RT-PCR was performed using the SYBR qPCR Master Mix fluorescent quantitation kit. Each gene was repeated three times, and the specificity of each primer pair was checked using melting curve analysis. *β-actin* was used as the reference gene [12]. The relative gene expression levels were analyzed using the 2^−ΔΔCt^ method [13]. 

### 2.5. Determination of Enzymatic Activities Related to Energy Metabolism

**Figure 2:**
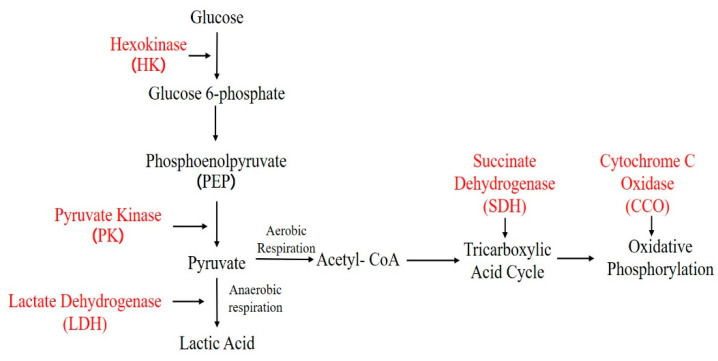
Flow chart of glucose metabolism. Note: The text marked in red indicates the enzyme activity detected in this study.

Enzymes related to energy metabolism, including cytochrome c oxidase (CCO), hexokinase (HK), succinate dehydrogenase (SDH), lactate dehydrogenase (LDH), and pyruvate kinase (PK), were detected in this study (Figure 2). The CCO activity assay was slightly modified based on the method of Liu et al. [14], and the oxidation of reduced cytochrome c was measured at a wavelength of 550 nm. The HK assay method was carried out based on the previous method reported by Eleazar [15]. SDH, LDH, and PK were determined using test kits purchased from Nanjing Jiancheng Bioengineering Institute. 

### 2.6. Transcriptome Analysis

Energy-metabolism-related pathway and differentially expressed gene (DEG) analyses were performed using transcriptome data previously obtained by our lab [16]. In particular, the changes in the oxidative phosphorylation pathway in EHP-infected shrimp were considered.

### 2.7. Mitochondrial Membrane Potential and Apoptosis

To investigate the effects of EHP on the *L. vannamei* mitochondrial function in relation to glucose metabolism, analyses of mitochondrial membrane potential and cell apoptosis were conducted in both healthy and infected groups. The hepatopancreas of *L. vannamei* was taken, and 2.5 g tissue was homogenized with 20 mL isolation buffer (320 mM sucrose, 10 mM Tris, 10 mM EGTA, 0.5% BSA, pH 7.3) at the lowest speed for 30 s. Then, the homogenate was centrifuged at 800 g for 10 min, and the supernatant was collected at 12,000 g for 15 min to precipitate the mitochondria. The obtained mitochondrial pellet was suspended in 0.25 mL of isolation buffer for subsequent mitochondrial characterization analysis. All the above operations were carried out on ice except for centrifugation at 4 °C. The protein concentration of the mitochondria was determined using the BCA reagent. Then, the purified mitochondria were used, and the mitochondrial membrane potential was detected using the JC-1 enhanced mitochondrial membrane potential detection kit (Beyotime). When the mitochondrial membrane potential is low, JC-1 exists as a monomer, whereas when the mitochondrial membrane potential is high, JC-1 will reaggregate in the mitochondrial matrix to form a polymer. Then, the relative ratio of monomers to polymers is often used to measure changes in mitochondrial membrane potential. 

At the same time, single-cell suspensions were prepared using the hepatopancreas of *L. vannamei*. After being filtered with a 100 μm mesh and resuspended in complete culture medium, a single-cell suspension was used to explore the cell apoptosis rate with the Annexin V-FITC apoptosis detection kit (Beyotime). Specifically, the hepatopancreatic cells were stained with FITC and PI and incubated in the dark for 15 min at room temperature, and then the cell apoptosis of *L. vannamei* in the healthy and infected groups was detected using a flow cytometer.

### 2.8. Statistical Analysis

The experimental data were collated by Microsoft Excel, 2021 and the experimental results were statistically analyzed by SPSS Statistics 25.0 (one-way ANOVA), and the Duncan method was used for multiple comparisons between groups. When *p* < 0.05, the difference was statistically significant.

## 3. Results

### 3.1. Ultrastructural Pathology 

**Figure 3:**
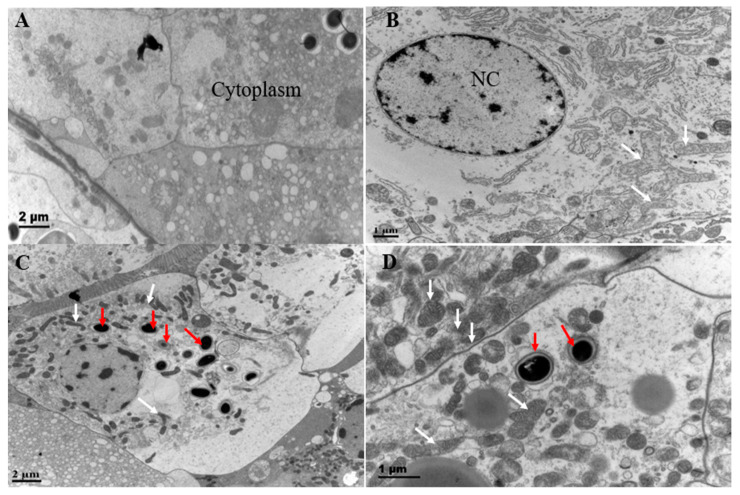
Histopathological analysis of hepatopancreatic tissues at different concentrations of EHP infection by transmission electron microscopy. Notes: (**A**,**B**) represent low-concentration EHP infection groups; (**C**,**D**) represent high-concentration EHP infection groups; NC means cell nucleus; red arrows represent EHP; white arrows represent mitochondria.

To observe ultrastructural pathological characteristics in tissues, transmission electron microscopy (TEM) analysis of hepatopancreas in shrimp naturally infected with EHP was performed. EHP spores, approximately 1 μm in size, were present in the cytoplasm of the hepatopancreas (Figure 3). In the high-concentration EHP infection group, the number of mitochondria increased significantly and showed obvious aggregation (Figure 3C,D). In the low-concentration EHP infection group, mitochondrial staining was lighter and more extended (Figure 3B).

### 3.2. Gene Expression Analysis

**Figure 4:**
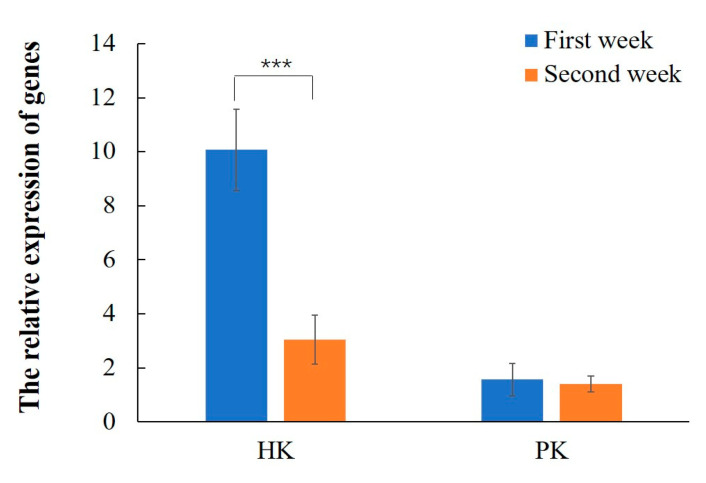
HK and PK gene expression in the hepatopancreas of *L. vannamei*. Notes: HK represents the hexokinase gene; PK represents the pyruvate kinase gene; *** means *p* ≤ 0.001.

In the EHP artificial infection experiment, quantitative results showed EHP content in the second week was 185-fold higher than that in the first week, which indicated the EHP content increased significantly with the increase in infection time. Fluorescence quantitative results of gene expression show that the key gene hexokinase was significantly upregulated in the diseased shrimp (Figure 4). The relative expression level of hexokinase in the first week was higher than that in the second week (*p* ≤ 0.001).

### 3.3. Energy-Metabolism-Related Enzyme Detection

**Figure 5:**
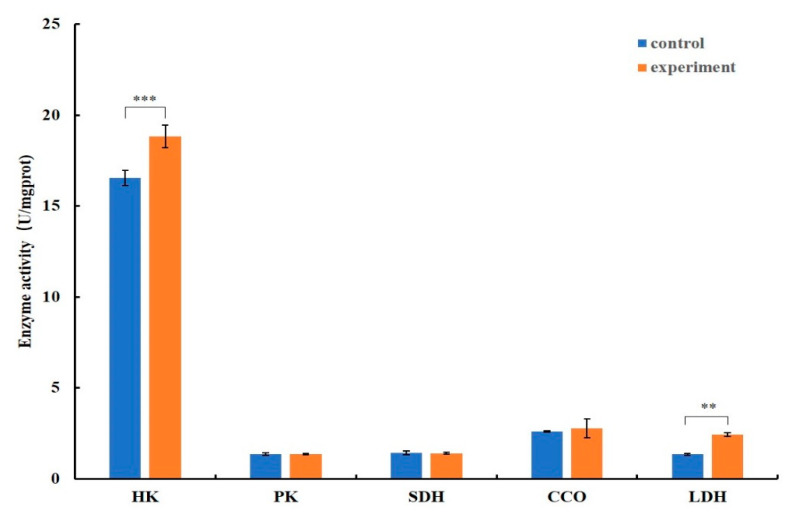
Enzyme activity analysis in the infected shrimp in the first week. Notes: The control group represents the healthy group (orange ) and the experiment group represents the artificial infection group (blue); ** means *p* ≤ 0.01, *** means *p* ≤ 0.001; HK is hexokinase, PK is pyruvate kinase, SDH is succinate dehydrogenase, CCO is cytochrome c oxidase, and LDH is lactate dehydrogenase.

After a week of infection, the hexokinase and lactate dehydrogenase activities of the hepatopancreas were significantly increased in the EHP-infected group. Meanwhile, the activity of the pyruvate dehydrogenase was slightly increased, but the difference was not significant. There was no significant change in the activities of cytochrome C oxidase and succinate dehydrogenase (Figure 5).

**Figure 6:**
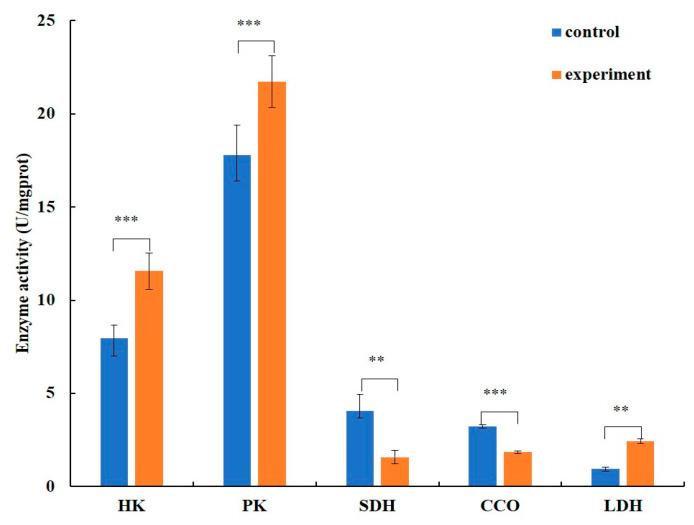
Enzyme activity analysis in the infected shrimp in the second week. Notes: The relevant annotations are consistent with those in Figure 5; ** means *p* ≤ 0.01, *** means *p* ≤ 0.001.

After two weeks of EHP infection, compared with the healthy group, the activities of hexokinase, pyruvate kinase, and lactate dehydrogenase in the hepatopancreas of shrimp were significantly increased in the EHP infection group, while the activities of succinate dehydrogenase and cytochrome C oxidase were significantly decreased (Figure 6).

### 3.4. EHP Effects on Energy Metabolism of L. vannamei Based on Transcriptome

**Figure 7:**
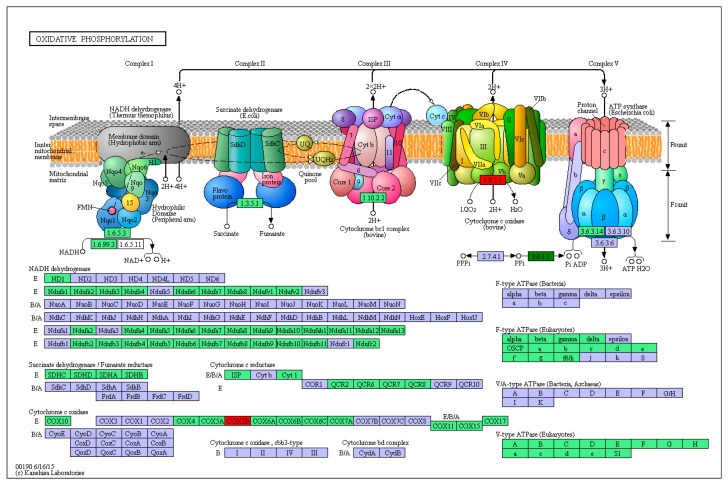
DEGs involved in oxidative phosphorylation pathway in group C vs. group A. Notes: Red indicates upregulated DEGs; dark green represents downregulated DEGs.

The Illumina sequencing and quality assessment, analysis of DEGs, and validation analysis of transcriptomics can be found in previously published literature [16]. Based on the transcriptome data, this article focuses on analyzing the energy-metabolism-related genes and signaling pathways of low-concentration and high-concentration EHP-infected groups. The KEGG analyses of differentially expressed genes (DEGs) between the low-concentration EHP-infected group (group C) and the high-concentration EHP-infected group (group A) were classified into five branches: metabolism, cellular processes, environmental information processing, genetic information processing, and organismal systems. Among these five branches, metabolism was the most enriched information pathway for the DEGs, including amino acid metabolism, carbohydrate metabolism, lipid metabolism, energy metabolism, and metabolism of other amino acids. The DEGs related to energy metabolism are shown in Table 2. Among them, *Cox5b* was the most significantly downregulated in high-concentration EHP infection, while Inorganic pyrophosphatase was the most significantly upregulated gene (Figure 7). These findings indicate that high-concentration EHP infection suppresses the oxidative phosphorylation pathway by *Cox5b*.

### 3.5. Mitochondrial Membrane Potential Changes

Mitochondrial membrane potential changes were estimated using JC-1 staining methods. In the first week, the ratio of monomers to polymers in the mitochondria of EHP-infected *L. vannamei* was significantly higher than that in the healthy group, indicating a significant decrease in mitochondrial membrane potential in the infected group. However, in the second week, the mitochondrial membrane potential still decreased to a certain extent but had no significant difference (Table 3). 

### 3.6. Hepatopancreas Cell Apoptosis

**Figure 8:**
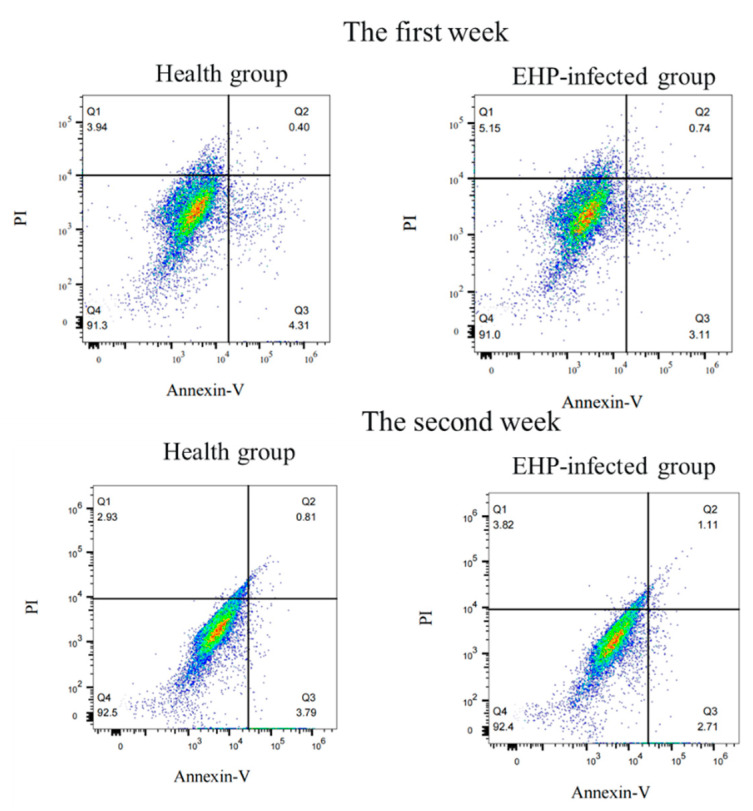
The analysis of cell apoptosis using flow cytometry.

During the first and second weeks of infection, primary hepatopancreas cells were isolated from both infected and healthy groups of shrimp to detect the level of cell apoptosis. The results showed no significant difference in cell apoptosis in the shrimp infected with EHP during the first and second weeks (Figure 8).

## 4. Discussions

EHP is a very serious pathogen in shrimp culture. However, due to the unique intracellular parasitic features, the complex pathogenicity, and unclear invasion mechanisms, there is still a lack of effective treatment and prevention measures for EHP. In this study, the pathogenic mechanism of EHP infection in hosts based on energy flow has been explored.

Ultrastructural pathological characteristics in the hepatopancreas of shrimp showed the number of mitochondria increased significantly and showed obvious aggregation in the high-concentration EHP infection group. However, in the low-concentration EHP infection group, mitochondrial staining was lighter and more extended. In *L. vannamei*, mitochondria are responsible for synthesizing ATP molecules required for cell function, providing sufficient energy to support life activities [17]. The increase in mitochondria in the EHP-infected shrimp means that the body needs more energy for the EHP pathogen and host.

In addition to their function in energy metabolism, mitochondria also participate in functions such as free radical production, aging, and cell apoptosis [18]. The changes in mitochondrial morphology under electron microscopy also indicated that EHP may affect not only host energy metabolism but also other functions of the host. Moreover, mitochondrial membrane potential plays a key role in maintaining normal cellular metabolism. The potential difference between the inner and outer mitochondrial membranes is an important driving force for ATP synthesis and affects cell metabolism and energy status. A decrease in the mitochondrial membrane potential can lead to an imbalance in cellular energy metabolism [19]. In this study, the mitochondrial membrane potential of the infected group was significantly lower than that of the healthy group in the first week of EHP infection, and there was no significant difference in the second week of EHP infection, implying that early EHP infection leads to mitochondrial damage and that the mitochondria were partially restored in the late stage. It is speculated that during the acute infection period of EHP, invasion and proliferation of EHP within cells may lead to damage in mitochondrial function in the hepatopancreas, while in the second week, although the mitochondrial membrane potential partially recovered, it still showed a certain degree of difference compared to the healthy group. The results of cell apoptosis showed that EHP infection had no significant effect on the apoptosis of hepatopancreas cells, indicating that EHP infection does not cause significant cell apoptosis, consistent with the conclusion that EHP does not directly cause death in *L. vannamei*.

In transcriptomic data analysis, energy-metabolism-related pathways revealed 13 DEGs associated with energy metabolism pathways. *Cox5b* encodes the peripheral nuclear-encoded subunit of mitochondrial cytochrome c oxidase subunit 5B, which is part of a multi-subunit, dual-genome-encoded protein complex catalyzing the final step of the mitochondrial electron transport chain. Previous studies have shown that *Cox5b* deficiency leads to a reduction in cytochrome c oxidase activity, indicating that *Cox5b* has a regulatory role [20]. Mitochondrial proteins play a critical role in the respiratory chain, and the downregulation of protein genes can induce mitochondrial dysfunction [21]. This gene is significantly downregulated in the high infection group of EHP, indicating that high concentrations of EHP infection can inhibit *Cox5b* gene expression and, therefore, suppress mitochondrial cytochrome c oxidase activity. Inorganic pyrophosphatase can catalyze chemical reactions to produce some important phosphate substances, such as ATP, NADH, FADH, and other compounds, which are extremely important biological energy sources. It can be seen that inorganic pyrophosphatase plays a huge role in maintaining normal metabolism and operation of organisms [22,23]. In addition, the *tpi1b* gene (Triosephosphate isomerase B) encodes a metabolic enzyme involved in the glycolytic pathway. In the infection group of EHP, inorganic pyrophosphatase and *tpi1b* were significantly upregulated, suggesting that EHP may enhance the host’s glycolytic pathway, accelerate glucose metabolism, and generate ATP.

In the enzyme activity and gene expression analysis, the glycolysis pathway and anaerobic respiration-related enzyme activities were higher than those of the healthy group in the first week of EHP infection, indicating that the glycolysis pathway and anaerobic respiration were activated. In the second week of EHP infection, the glycolysis pathway and anaerobic respiration were still activated, while the tricarboxylic acid cycle and oxidative phosphorylation levels decreased. The quantitative gene expression results in this study were consistent with the enzyme activity detection results. When subjected to environmental stress and other unfavorable conditions, *L. vannamei* switches to anaerobic energy metabolism and activates its enzymatic pathways, which promotes the generation of ATP [24]. In response to EHP infection, *L. vannamei* exhibits an activated state of glycolysis in the first and second weeks, and it is speculated that *L. vannamei* may enhance its tolerance to EHP by increasing the rate of glycolysis, thereby leading to a reduction in energy consumption for growth; this is possibly the key reason for its growth retardation. Lactate dehydrogenase (LDH) catalyzes the bidirectional conversion of lactate to pyruvate and is a glycolytic enzyme that provides additional energy supply when the organism is under stress [25]. In this study, the LDH activity was significantly increased during the first and second weeks of EHP infection, resulting in an increase in the conversion of pyruvate to lactate and enhancing anaerobic metabolism in tissues. Many cancer cells exhibit a preference for glycolysis, even in the presence of oxygen, which manifests as high levels of glucose uptake and lactate production [26]. The Warburg effect is an abnormal glycolysis response that is associated with cancer cells. This preference is due to changes and abnormal activation in gene expression and signaling pathways. Some scholars have proposed metabolic changes resembling the Warburg effect are induced by a nonmammalian virus [27,28]. In this paper, we propose that EHP also induces this effect for the first time, which is beneficial for EHP self-growth. After EHP infection, the energy metabolism pattern of *L. vannamei* is similar to that of cancer cells, with an increase in glycolysis and anaerobic respiration and a suppression of oxidative phosphorylation and aerobic respiration. This metabolic shift may be due to EHP’s energy theft in the host leading to abnormal gene expression and signaling pathways in *L. vannamei*.

Based on the pathological observations, enzyme activity, and gene expression analysis of *L. vannamei*, as well as a transcriptomic analysis, it is indicated that EHP infection can lead to abnormal mitochondrial energy supply, evidenced by the increase in and aggregation of mitochondria, as well as the activation of the glycolytic pathway and inhibition of the oxidative phosphorylation pathway.

## Figures and Tables

**Table 1:** Oligonucleotide primers.

Gene Name	Primer	Primer Sequence
*β-actin*	β-actin-F	CCGGCCGCGACCTCACAGACT
	β-actin-R	CCTCGGGGCAGCGGAACCTC
*HK*	HK-F	AGTCGCAGCAACAGGAAGTT
	HK-R	CGCTCTTCTGGCACATGATA
*PK*	PK-F	AGCTGTCTCAGCAGGTCCAT
	PK-R	AGCGAGGCCTGTACTTTGAA

**Table 2:** DEGs enriched in energy metabolism pathway in group C vs. group A.

Gene ID	Gene Name	Description	Fold Change
LOC113799962	*DPYD*	Dihydropyrimidine dehydrogenase [NADP(+)]	2.40
LOC113800564	*PHGDH*	D-3-phosphoglycerate dehydrogenase	8.34
LOC113801308	*CG11899*	Probable phosphoserine aminotransferase	8.60
LOC113802550	*tpi1b*	Triosephosphate isomerase B	0.08
LOC113803894	*-*	Glutamine synthetase	2.50
LOC113805920	*Acss1*	Acetyl-coenzyme A synthetase 2-like, mitochondrial	0.37
LOC113812240	*MTHFR*	Methylenetetrahydrofolate reductase	5.72
LOC113815714	*PGAM2*	Phosphoglycerate mutase 2	0.18
LOC113816682	*Psph*	Phosphoserine phosphatase	3.19
LOC113823145	*-*	Glutamine synthetase	0.31
LOC113826894	*CG11899*	Probable phosphoserine aminotransferase	9.02
LOC113829904	*-*	Inorganic pyrophosphatase	0.030
LOC113827783	*Cox5b*	Cytochrome c oxidase subunit 5B, mitochondrial	64.09

**Table 3:** Mitochondrial membrane potential detection based on JC-1.

Monomer/Polymer	Healthy Group	EHP-Infected Group	*p* Value
The first week	0.086 ± 0.0015	0.290 ± 0.0032	*
The second week	0.043 ± 0.0012	0.062 ± 0.0014	-

Notes: * means *p* ≤ 0.05, - means *p* > 0.05.

## Data Availability

The raw data supporting the conclusions of this article will be made available by the authors, without undue reservation. The use of experimental animals was reviewed and approved by the ethics committee of Ningbo University.

## References

[1] Farzanfar A. (2010). The use of probiotics in shrimp aquaculture. Pathog. Dis..

[2] Ma R., Wu Y., Li G., Zhao S., Li L., Fang W. (2022). Pharmacokinetics of sarafloxacin hydrochloride in the pacific white shrimp, *Litopenaeus vannamei*, following multiple-dose oral administration. Aquaculture.

[3] Jaroenlak P., Boakye D.W., Vanichviriyakit R., Williams B.A.P., Sritunyalucksana K., Itsathitphaisarn O. (2018). Identification, characterization and heparin binding capacity of a spore-wall, virulence protein from the shrimp microsporidian, *Enterocytozoon hepatopenaei* (EHP). Parasites Vectors.

[4] Tourtip S., Wongtripop S., Stentiford G.D., Bateman K.S., Sriurairatana S., Chavadej J., Sritunyalucksana K., Withyachumnarnkul B. (2009). Enterocytozoon hepatopenaei sp. nov. (Microsporida: Enterocytozoonidae), a parasite of the black tiger shrimp *Penaeus monodon* (Decapoda: Penaeidae): Fine structure and phylogenetic relationships. J. Invertebr. Pathol..

[5] Tang K.F., Han J.E., Aranguren L.F., White-Noble B., Schmidt M.M., Piamsomboon P., Risdiana E., Hanggono B. (2016). Dense populations of the microsporidian *Enterocytozoon hepatopenaei* (EHP) in feces of *Penaeus vannamei* exhibiting white feces syndrome and pathways of their transmission to healthy shrimp. J. Invertebr. Pathol..

[6] Jaroenlak P., Sanguanrut P., Williams B.A.P., Stentiford G.D., Flegel T.W., Sritunyalucksana K., Itsathitphaisarn O. (2016). A Nested PCR Assay to Avoid False Positive Detection of the Microsporidian *Enterocytozoon hepatopenaei* (EHP) in Environmental Samples in Shrimp Farms. PLoS ONE.

[7] Liu Y.M., Qiu L., Sheng A.Z., Wan X.Y., Cheng D.Y., Huang J. (2018). Quantitative detection method of *Enterocytozoon hepatopenaei* using TaqMan probe real-time PCR. J. Invertebr. Pathol..

[8] Nakjang S., Williams T.A., Heinz E., Watson A.K., Foster P.G., Sendra K.M., Heaps S.E., Hirt R.P., Embley T.M. (2013). Reduction and Expansion in Microsporidian Genome Evolution: New Insights from Comparative Genomics. Genome Biol. Evol..

[9] Hacker C., Howell M., Bhella D., Lucocq J. (2014). Strategies for maximizing ATP supply in the microsporidian Encephalitozoon cuniculi: Direct binding of mitochondria to the parasitophorous vacuole and clustering of the mitochondrial porin VDAC. Cell. Microbiol..

[10] Boakye D.W., Jaroenlak P., Prachumwat A., Williams T.A., Bateman K.S., Itsathitphaisarn O., Sritunyalucksana K., Paszkiewicz K.H., Moore K.A., Stentiford G.D. (2017). Decay of the glycolytic pathway and adaptation to intranuclear parasitism within Enterocytozoonidae microsporidia. Environ. Microbiol..

[11] Luo Y., Shi J., Fang L., Meng Q., Zhang X., Qian D. (2016). Development and application of a TaqMan real-time PCR assay for the detection of Enterocytozoon hepatopenaei. Chin. Vet. Sci..

[12] Zhang X., Yang H., Li H., Chen T., Ruan Y., Ren C., Luo P., Wang Y., Liu B., Li H. (2021). Molecular Identification of Anion Exchange Protein 3 in Pacific White Shrimp. Front. Physiol..

[13] Livak K.J., Schmittgen T.D. (2001). Analysis of relative gene expression data using real-time quantitative PCR and the 2^(−∆∆C^_T_^)^ Method. Methods.

[14] Liu Z.M., Wang G.Z., Li S., Chen Z. (2018). Mitochondrial Respiration Rate and Enzyme Activity of Two Populations of *Scylla paramamosain* During Low Temperature Seasons. J. Xiamen Univ. (Nat. Sci.).

[15] Martinez-Barajas E., Randall D.D. (1998). Purification and characterization of a glucokinase from young tomato (*Lycopersicon esculentum* L. Mill.) fruit. Planta.

[16] Zhu B., Lu X., Liu Y., Wu Z., Cai H., Jin S., Li Z., Xie J., Li X., Sun F. (2022). Effects of *Enterocytozoon hepatopenaei* single-infection or co-infection with *Vibrio parahaemolyticus* on the hepatopancreas of *Penaeus vannamei*. Aquaculture.

[17] Li T., Li E., Suo Y., Xu Z., Jia Y., Qin J.G., Chen L., Gu Z. (2017). Energy metabolism and metabolomics response of Pacific white shrimp Litopenaeus vannamei to sulfide toxicity. Aquat. Toxicol..

[18] Zamzami N., Susin S.A., Marchetti P., Hirsch T., Kroemer G. (1996). Mitochondrial control of nuclear apoptosis. J. Exp. Med..

[19] Mendez-Romero O., Uribe-Carvajal S., Chiquete-Felix N., Muhlia-Almazan A. (2019). Mitochondrial uncoupling proteins UCP4 and UCP5 from the Pacific white shrimp *Litopenaeus vannamei*. J. Bioenerg. Biomembr..

[20] Hu T., Xi J. (2017). Identification of COX5B as a novel biomarker in high-grade glioma patients. Oncotargets Ther..

[21] Calvo S.E., Mootha V.K. (2010). The mitochondrial proteome and human disease. Annu. Rev. Genom. Hum. Genet..

[22] Hughes R.C., Coates L., Blakeley M.P., Tomanicek S.J., Langan P., Kovalevsky A.Y., García-Ruiz J.M., Ng J.D. (2012). Inorganic pyrophosphatase crystals from Thermococcus thioreducens for X-ray and neutron diffraction. Acta Crystallogr. Sect. F.

[23] Salminen T., Kaepylae J., Heikinheimo P., Kankare J., Goldman A., Heinonen J., Baykov A.A., Cooperman B.S., Lahti R. (1995). Structure and function analysis of Escherichia coli inorganic pyrophosphatase: Is a hydroxide ion the key to catalysis?. Biochemistry.

[24] Gao X., Li X., Shi C., Wu F., Song C., Liu Y. (2018). Effects of stocking density on growth, metabolism, and energy budget of Haliotis discus hannai Ino. Aquaculture.

[25] Xu C., Li E., Liu Y., Wang X., Qin J.G., Chen L. (2017). Comparative proteome analysis of the hepatopancreas from the Pacific white shrimp Litopenaeus vannamei under long-term low salinity stress. J. Proteom..

[26] Han M., Bushong E.A., Segawa M., Tiard A., Wong A., Brady M.R., Momcilovic M., Wolf D.M., Zhang R., Petcherski A. (2023). Spatial mapping of mitochondrial networks and bioenergetics in lung cancer. Nature.

[27] Chen I.T., Aoki T., Huang Y.T., Hirono I., Wang H.C. (2011). White spot syndrome virus induces etabolic changes resembling the Warburg effect in shrimp hemocytes in the early stage of infection. J. Virol..

[28] Apún-Molina J.P., Robles-Romo A., Alvarez-Ruiz P., Santamaria-Miranda A., Arjona O., Racotta I.S. (2017). Influence of stocking density and exposure to white spot syndrome virus in biological performance, metabolic, immune, and bioenergetics response of whiteleg shrimp *Litopenaeus vannamei*. Aquaculture.

